# Supraglottic airway devices as a strategy for unassisted tracheal intubation: A network meta-analysis

**DOI:** 10.1371/journal.pone.0206804

**Published:** 2018-11-05

**Authors:** EunJin Ahn, GeunJoo Choi, Hyun Kang, ChongWha Baek, YongHun Jung, YoungCheol Woo, SiRa Bang

**Affiliations:** 1 Department of Anaesthesiology and Pain Medicine, InJe University Seoul Paik Hospital, Seoul, Korea; 2 Department of Anaesthesiology and Pain Medicine, Chung-Ang University College of Medicine, Seoul, Korea; San Gerardo Hospital, ITALY

## Abstract

We aimed to compare the effectiveness of supraglottic airway devices as a strategy for unassisted tracheal intubation. Accordingly, we searched the OVID-MEDLINE, EMBASE, Cochrane Central Register of Controlled Trials, KoreaMed, and Google Scholar databases to identify all relevant randomized controlled trials (RCTs) on supraglottic airway devices as a strategy for tracheal intubation published until May 2017. The primary outcome was the overall success rate of intubation by the intention to treat (ITT) strategy. The secondary outcomes of the study were the overall success rate of tracheal intubation by the per protocol (PP) strategy and the success rate of tracheal intubation at first attempt by ITT and PP. We conducted a network meta-analysis with a mixed-treatment comparison method to combine direct and indirect comparisons among supraglottic airway devices. Of 1396 identified references, 16 RCTs (2014 patients) evaluated unassisted intubation with supraglottic airway devices. Patients were grouped according to the type of device used: LMA-CTrach, LMA-Fastrach, Air-Q, i-gel, CobraPLA, Ambu-Aura, or single-use LMA devices. Based on the surface under the cumulative ranking curve, the three best supraglottic airway devices for use as a strategy for unassisted tracheal intubation were LMA-CTrach (which included video-assisted tracheal tube guidance), single-use LMA-Fastrach, and LMA-Fastrach. LMA-Fastrach showed a higher success rate of intubation than did i-gel, CobraPLA, Air-Q, and Ambu-Aura. However, this study was limited by the small number of eligible RCTs. Therefore, well-designed RCTs performed on large patient populations are required to increase the confidence of the results.

## Introduction

Tracheal intubation is considered a gold standard for airway maintenance, which prevents pulmonary aspiration with stable ventilation. For tracheal intubation, Macintosh direct laryngoscopy is the gold standard. However, owing to the high failure rate of the direct laryngoscope (up to 30%)[1], several devices including the videolaryngoscope and supraglottic airway (SGA) devices have been developed[2, 3]. SGA devices designed for unassisted tracheal intubation have also been developed. Furthermore, endotracheal intubation using SGA devices is a widely investigated and accepted technique that has been recognized on the emergency pathway of the American Society of Anesthesiologists’ Difficult Airway Algorithm[2].

Many devices are available for performing endotracheal intubation, such as the videolaryngoscope, intubating SGA device, and direct laryngoscope. Among them, the videolaryngoscope provides indirect close-proximity glottic visualization, which increases the success rate of intubation at first attempt and enables faster intubation[4]. Unlike the videolaryngoscope, SGA devices are used to perform unassisted tracheal intubation. Moreover, SGA devices designed for tracheal intubation enable ventilation and provide a strategy for unassisted tracheal intubation in patients with emergency airway compromise (i.e., cannot ventilate status).

Intubation through SGA devices can be performed either under fiberoptic guidance or unassisted. Intubation is considered “unassisted” when the tube is inserted through the SGA device without the assistance of other devices within the larynx or pharynx. The success rate of unassisted intubation ranges widely from 15% to 97%, depending on the type of SGA device, patient characteristics, and operator skill [5–8]. Among SGA devices analyzed in our study, only LMA-CTrach allows for the visualization of the vocal cords by using its built-in fiberoptic system. Furthermore, some randomized controlled trials (RCTs) have reported that the type and curvature of the endotracheal tube affect the success rate of unassisted intubation[9, 10].

Therefore, we reviewed articles that compared the success rate of unassisted intubation between SGA devices and performed a statistical analysis via a network meta-analysis (NMA) to compare the effectiveness of SGA devices as a strategy for unassisted tracheal intubation.

## Materials and methods

We developed the protocol for this review and registered it on the PROSPERO network (registration number: CRD42017051153; www.crd.york.ac.uk/PROSPERO) on May 15, 2017. The present systematic review and meta-analysis was conducted according to the protocol recommended by the Cochrane Collaboration[11] and presented following the Preferred Reporting Items for Systematic Reviews and Meta-Analyses guidelines for reporting a NMA[12].

### Inclusion criteria

We included only RCTs that compared SGA devices as a strategy for unassisted tracheal intubation. The inclusion criteria were as follows:

1. RCTs,
2. patients who underwent surgery under general anesthesia by using SGA devices as a strategy for tracheal intubation without the use of fiberoptic bronchoscopic guidance, and
3. comparison between two or more different types of SGA device groups.

### Exclusion criteria

RCTs that only investigated SGA devices as a strategy for fiberoptic-guided tracheal intubation were excluded.

### Information sources and search strategy

Two authors (HK and EJA) independently searched the OVID-MEDLINE, EMBASE, Cochrane Central Register of Controlled Trials (CENTRAL), KoreaMed (http://www.koreamed.org), and Google Scholar databases for all relevant articles from the inception of the databases through November 2016 and updated in May 2017 (1946 to present in OVID-MEDLINE, 1974 to present in EMBASE, current issue in CENTRAL, 1996 to present in KoreaMed, and 1990 to present on Google Scholar). The search strategy, which included a combination of free text, Medical Subject Headings, and EMTREE terms, is described in the supplemental digital content.

### Study selection

Two authors (EJA and GJC) independently scanned the titles and abstracts of the reports identified via the search strategies described above. If a report was determined to be potentially eligible from the title or abstract by either author, the full paper was retrieved and the full-text version was evaluated. RCTs were included or excluded on the basis of consensus between the two authors (EJA and GJC); any disagreement over inclusion or exclusion was settled in discussion with a third investigator (HK).

### Data extraction

All interrelated data from the included RCTs were independently extracted and entered into a standardized form by two authors (CWB and YHJ), and then cross-checked. Any discrepancy was resolved through discussion. If an agreement could not be reached, the dispute was resolved with the aid of a third investigator (YCW). The standardized form included the following items: (1) title, (2) name of the first author, (3) name of the journal, (4) year of publication, (5) study design, (6) SGA devices used, (7) subject of study (adults vs. children), (8) country, (9) risk of bias, (10) inclusion criteria, (11) exclusion criteria, (12) sex, (13) age, (14) number of subjects, (15) overall success rate, and (16) success rate at first attempt.

The data were initially extracted from tables or text. In cases involving missing or incomplete data, an attempt was made to contact the study authors to obtain the relevant information.

### Risk of bias assessment

The quality of RCTs was independently assessed by two authors (GJC and EJA) by using the tool “risk of bias” according to Review Manager (version 5.3, The Cochrane Collaboration, Oxford, UK). Quality was evaluated using the following potential sources of bias: sequence generation, allocation concealment, blinding of participants or outcome assessor, incomplete data, and selective reporting. The methodology for each study was graded as “high,” “low,” or “unclear,” which reflected a high risk of bias, low risk of bias, and uncertain bias, respectively[11].

### Statistical analysis

Herein, we have briefly summarized our methodology because of space considerations; further details are available in our original protocol (supplementary file). A multiple-treatment comparison NMA is a generalization of meta-analysis methods that includes both direct RCT comparisons and indirect comparisons of treatments. A NMA was performed using STATA software (version 15; StataCorp LP, College Station, TX, USA) *mvmeta* with NMA graphical tools by Chaimani et al[13].

The primary outcome of this study was the overall success rate of intubation by the intention to treat (ITT) strategy. The secondary outcomes were the overall success rate of tracheal intubation by the per protocol (PP) strategy and the success rate of tracheal intubation at first attempt by ITT and PP.

For contribution assessment, we derived the direct estimates by using a comparison-specific random-effects model. We evaluated the consistency assumption for the entire network by using the design-by-treatment interaction model [14], and examined each closed loop in the network to evaluate local inconsistency between the direct and indirect effect estimates for the same comparison. Only triangular (formed by three treatments compared with one another) loops were considered. No quadratic loop was included in our network. In each loop, we estimated the inconsistency factor (IF) as the absolute difference (with 95% confidence interval [CI] and a z-test) between the direct and indirect estimates for each paired comparison in the loop. The IF is the logarithm of the ratio of two odds ratios (RoRs) from direct and indirect evidences in the loop; RoRs close to 1 indicate that the two sources are in agreement.

A comparison-adjusted funnel plot was used to assess the presence of the small-study effect[15]. The mean summary effects were presented together with their predictive intervals (PrIs) to facilitate the interpretation of results with respect to the magnitude of heterogeneity. PrIs provide an interval within which the estimate of a future study is expected to be.

We used the surface under the cumulative ranking curve (SUCRA) values to present the hierarchy of interventions for the overall success rate and success rate at first attempt. SUCRA is a relative ranking measure that accounts for the uncertainty in treatment order, i.e., it accounts for both the location and variance of all relative treatment effects[16]. A higher SUCRA value was regarded as a better result for individual intervention. A rankogram plots the probabilities for treatments to assume any of the possible ranks. Sensitivity analyses were performed by excluding the study performed in children[17].

## Results

**Fig 1:**
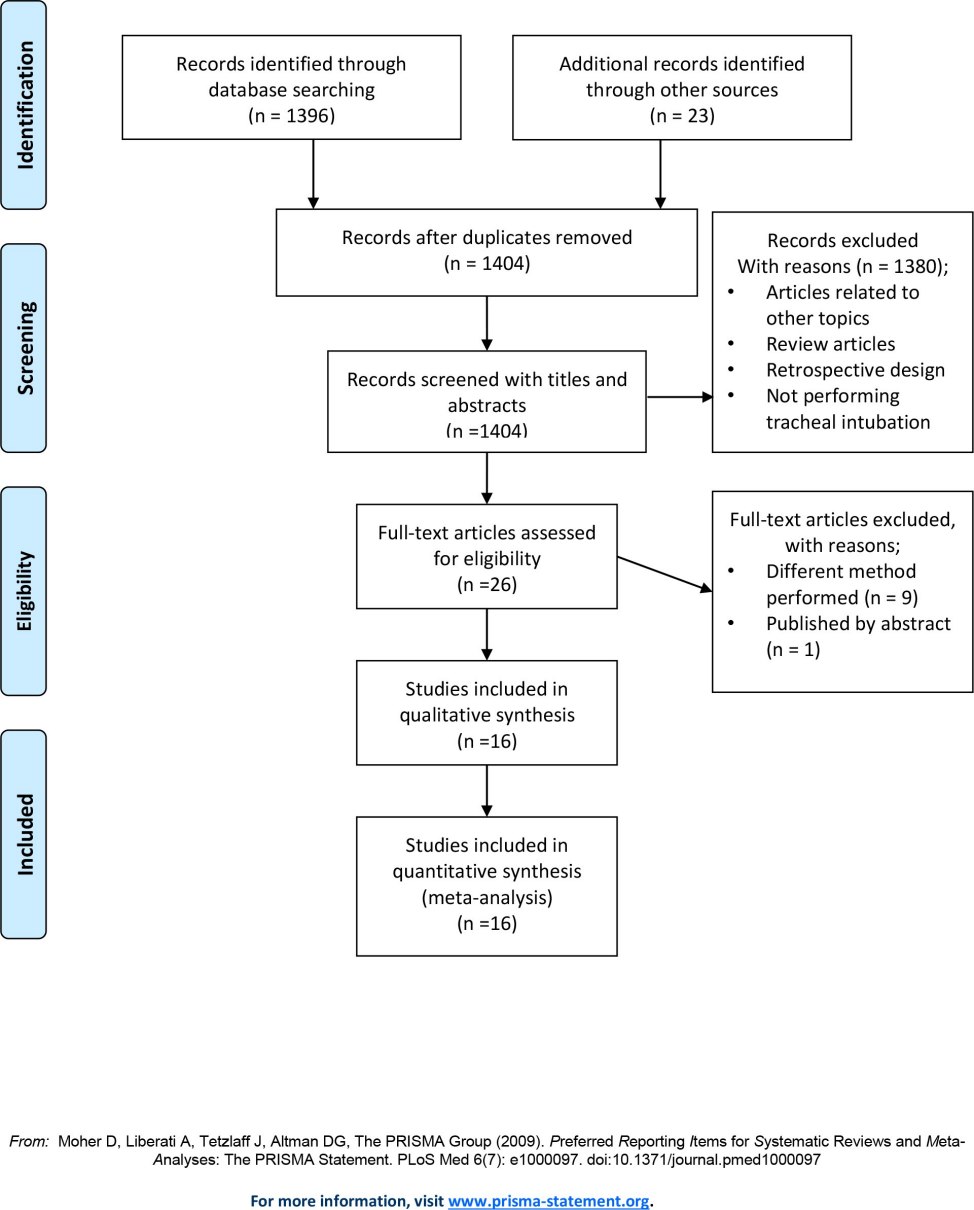
Flow diagram.

The searches of the OVID-MEDLINE, EMBASE, CENTRAL, KoreaMed, and Google Scholar databases initially identified 1396 studies, and the subsequent manual search revealed 23 additional studies. After adjusting for duplicates, 1404 studies remained. Of these, 1380 studies were excluded after reviewing the titles and abstracts because of the following reasons: studies were related to other topics, designed as reviews or retrospective studies, or did not perform tracheal intubation using SGA devices. The remaining 26 studies were reviewed in detail; nine studies were excluded because they only reported the results of fiberoptic-guided intubation[18–26], and one study was excluded because it was a published abstract[27]. Thus, 16 RCTs including 2014 patients met the inclusion criteria and were included in this NMA (Fig 1)[6–8, 17, 28–39].

### Study characteristics

The characteristics of the 16 RCTs that met the inclusion criteria are summarized in Table 1 and Table 2. Among the 16 RCTs, only one was performed in pediatric patients[17]. Two RCTs were written in Spanish[29, 35] and one article was written in Chinese[39]; all other RCTs were written in English. Seven SGA devices were compared in the included RCTs (single-use LMA-Fastrach, CobraPLA, Air-Q, LMA-Fastrach, i-gel, Ambu-Aura, and LMA-CTrach). Two RCTs used Ambu-Aura SGA devices: Kleine-Brueggeney et al[17]. used Ambu Aura-I, and Sethi et al[37]. used Ambu AuraGain. However, we regarded Ambu Aura-I and Ambu AuraGain as one SGA under Ambu-Aura. Kleine-Brueggeney et al[17] included pediatric patients, and the remaining 15 RCTs included only adult patients.

**Table 1:** Baseline characteristics of the included randomized controlled trials.

Author, Year	Journal	Number of patients	Subject of study	Nation	Language
**Anuradha 2017**	Int J Sci Stud	80	Adults	India	English
**Darlong 2011**	Acta Anaesthesiol Taiwan	60	More than 15 years old	India	English
**Erlacher 2011**	Eur J Anaesthesiol	180	Adults	Austria	English
**Garzon 2014**	Rev Esp Anestesiol Reanim	80	Adults	Spain	Spanish
**Halwagi 2012**	Anesth Analg	160	Adults	Canada	English
**Kapoor 2014**	Indian J Anaesth	100	Adults	India	English
**Karim 2011**	Anaesthesia	154	Adults	USA	English
**Kleine 2015**	Eur J Anaesthesiol	80	Pediatric patients	Switzerland	English
**Liu 2008**	Anesthesiology	268	Adults	Singapore	English
**Malhotra 2016**	Indian J Anaesth	120	Adults	India	English
**Neoh 2012**	South Afr J Anaesth Analg	160	Adults	USA	English
**Sastre 2012**	Rev Esp Anestesiol Reanim	80	Adults	Spain	Spanish
**Sethi 2017**	Egyptian Journal of Anesthesia	90	Adults	India	English
**Teoh 2007**	Anaesthesia	84	Adults	Singapore	English
**Theiler 2011**	Br J Anaesth	80	Adults	Switzerland	English
**Yang 2013**	Zhongguo Yi Xue Ke Xue Yuan Xue Bao	60	Adults	China	Chinese

**Table 2:** Characteristics of the included randomized controlled trials.

Author, Year	Type of tube	Comparison of airway devices	Age	Mallampatti	1st	Overall	Total attempt
**Anuradha 2017**	PVC ETT	i-gel vs LMAF	29.17 (5.47)	1, 2	24/40 35/40	29/40 38/40	2
**Darlong 2011**	PVC ETT, silicone-wire-reinforced tube	CobraPLA vs LMAF	27 (11.9)	1, 2	19/30 21/30	26/30 27/30	3
**Erlacher 2011**	Silicone-reinforced tube, PVC ETT, PVC ETT	LMAF vs CobraPLA vs Air-Q	Not reported	1, 2, 3, 4	Not reported	57/60 28/60 35/60	3
**Garzon 2014**	Silicone-reinforced tube, PVC ETT	LMAF vs Air-Q	57.05 (17.66)	1, 2	24/38 14/32	30/40 24/40	2
**Halwagi 2012**	PVC ETT	i-gel vs single LMAF	53.8 (14.3)	1, 2, 3	55/77 59/80	59/80 73/80	3
**Kapoor 2014**	PVC ETT	i-gel vs LMAF	34.87 (10.61)	1, 2	33/50 37/50	41/50 48/50	2
**Karim 2011**	Reinforced tube, PVC ETT	Single-use LMAF vs Air-Q	51 (14)	Not reported	71/76 55/78	75/76 60/78	2
**Kleine 2015**	PVC ETT	Air-Q vs Aura-I	4.3 (1.9–7.4)	Not reported	Not reported	6/39 1/40	3
**Liu 2008**	Reinforced tube, not reported	LMAF vs LMA-CTrach	43.6 (14.1)	1, 2, 3, 4	93/137 125/134	132/137 134/134	3
**Malhotra 2016**	Reinforced tube, reinforced tube or PVC ETT	LMAF vs air-Q	41.7 (12.16)	1, 2	45/60 39/60	55/60 58/60	3
**Neoh 2012**	PVC ETT, silicone ETT	Air-Q vs LMAF	40.9 (12.0)	1, 2, 3	51/80 65/79	60/80 77/80	3
**Sastre 2012**	PVC ETT, silicone-reinforced tube	LMAF vs i-gel	57.05 (17.6)	Not reported	24/36 14/34	28/40 16/40	2
**Sethi 2017**	PVC ETT	Aura-gain vs Air-Q	33.2 (7.8)	Not reported	16/45 31/45	24/45 36/45	2
**Teoh 2007**	Not reported	Single-use LMAF vs LMAF	Not reported	1, 2	25/40 27/40	37/42 37/42	3
**Theiler 2011**	Reinforced tube, PVC ETT	Single use LMAF vs i-gel	57 (24–48)	1, 2, 3	27/39 6/39	27/39 6/39	1
**Yang 2013**	Not described	Air-Q vs LMAF	29.4 (13.0)	?	35/43 32/43	42/43 39/43	3

ETT = endotracheal tube; LMAF = LMA-Fastrach; PLA = perilaryngeal airway; PVC = polyvinyl chloride.

All RCTs had obtained ethical approval from their respective Institutional Review Boards. Five RCTs had been registered in the clinical trial registries (Sethi et al[37]. 2017, CTRI/2015/02/005553; Kleine-Brueggeney et al[17]. 2015, NCT01692522; Halwagi et al[31]. 2012, NCT01007370; Kapoor et al[32]. 2014, REF/2013/12/006091; and Theiler et al[8]. 2011, NCT00888875).

### Risk of bias assessment

The quality indicators of the included RCTs are described in supplementary file “S1 Table.” The most common risk was incomplete blinding of participants, which was defined as the difficulty in blinding personnel who inserted the different SGA devices in each patient. Therefore, we exempted this parameter when scoring the RCTs to be at an overall high risk of bias only when they also demonstrated a high risk of bias in at least one other domain. One study[7] was designated as having an overall high risk of bias, and six RCTs[8, 17, 35–38] were determined as having a low risk of bias. The remaining nine RCTs[6, 28–34, 39] had an unclear risk of bias.

### Synthesis of results

Before conducting the NMA, we evaluated the transitivity assumption by examining the comparability of the risk of bias as a potential treatment-effect modifier across comparisons. This suggested that the transitivity assumption was not violated.

**Fig 2:**
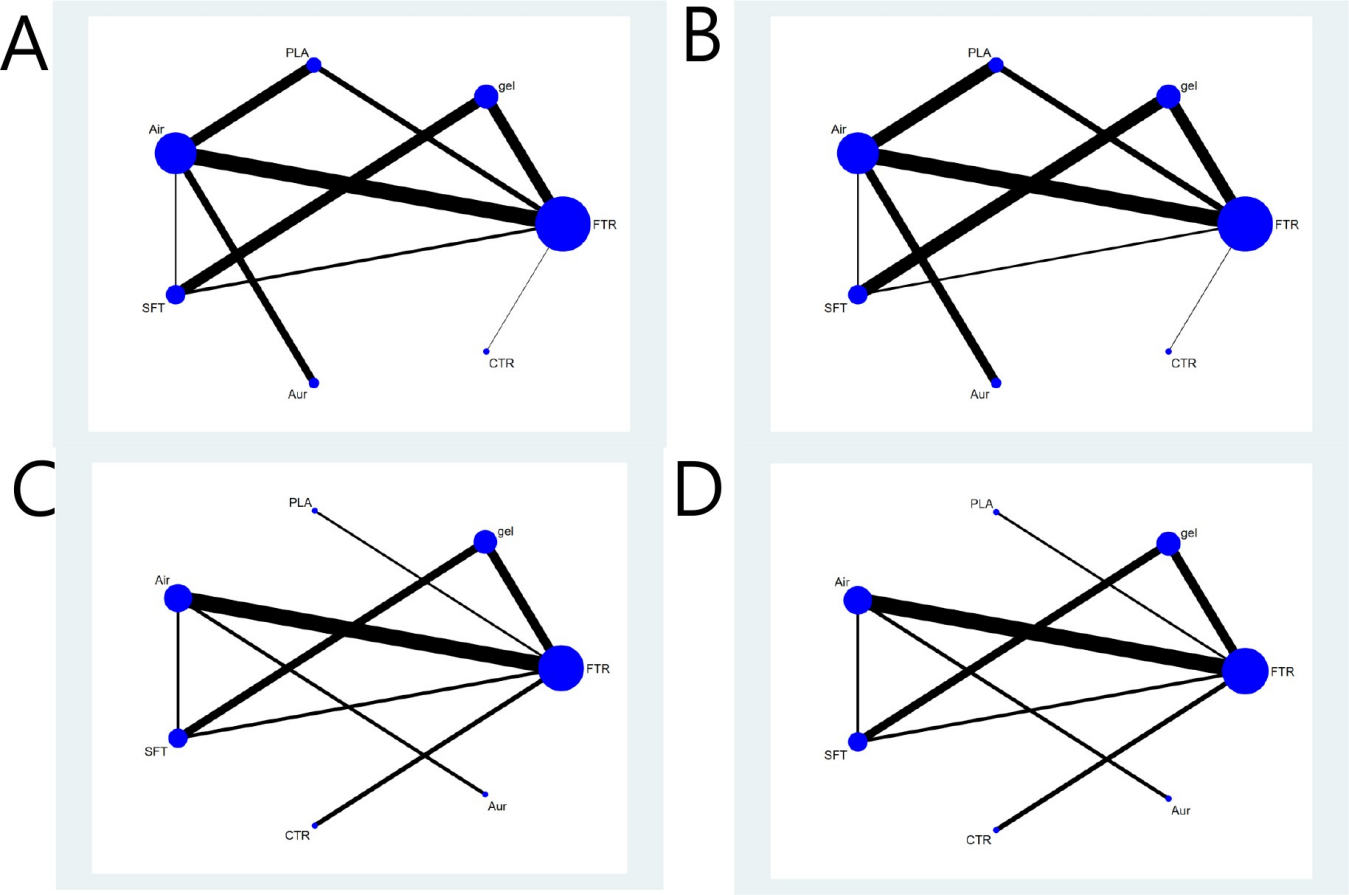
Network geometries. A network plot of direct comparisons for all included randomized controlled trials for the outcome of unassisted tracheal intubation. The width of the lines is proportional to the number of trials comparing each pair of treatments, and the size of every node is proportional to the number of randomized participants (sample size). A. The overall success rate of unassisted tracheal intubation by ITT; B. The overall success rate of unassisted tracheal intubation by PP; C. The success rate at first attempt by ITT; D. The success rate at first attempt by PP. ITT, intention to treat; PP, per protocol. SFT = single-use LMA-Fastrach; PLA = CobraPLA; Air = Air-Q; FTR = LMA-Fastrach; gel = i-gel; Aur = Ambu-Aura; CTR = LMA-CTrach.

**Fig 3:**
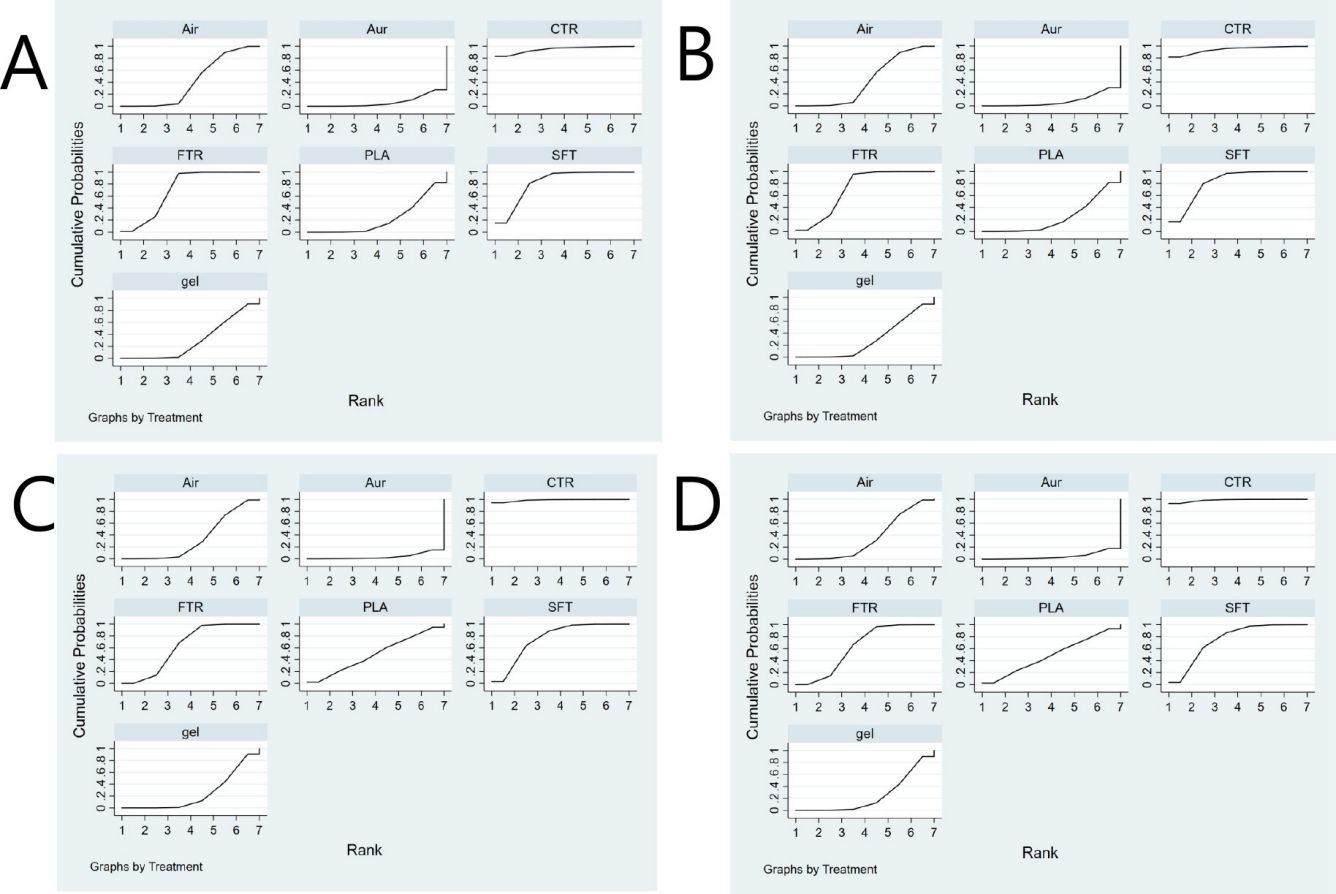
SUCRA probabilities. The SUCRA values of different supraglottic airway devices as a strategy for tracheal intubation. A. The overall success rate of unassisted tracheal intubation by ITT; B. The overall success rate of unassisted tracheal intubation by PP; C. The success rate at first attempt by ITT; D. The success rate at first attempt by PP. SUCRA, surface under the cumulative ranking curve; ITT, intention to treat; PP, per protocol. SFT = single-use LMA-Fastrach; PLA = CobraPLA; Air = Air-Q; FTR = LMA-Fastrach; gel = i-gel; Aur = Ambu-Aura; CTR = LMA-CTrach.

**Fig 4:**
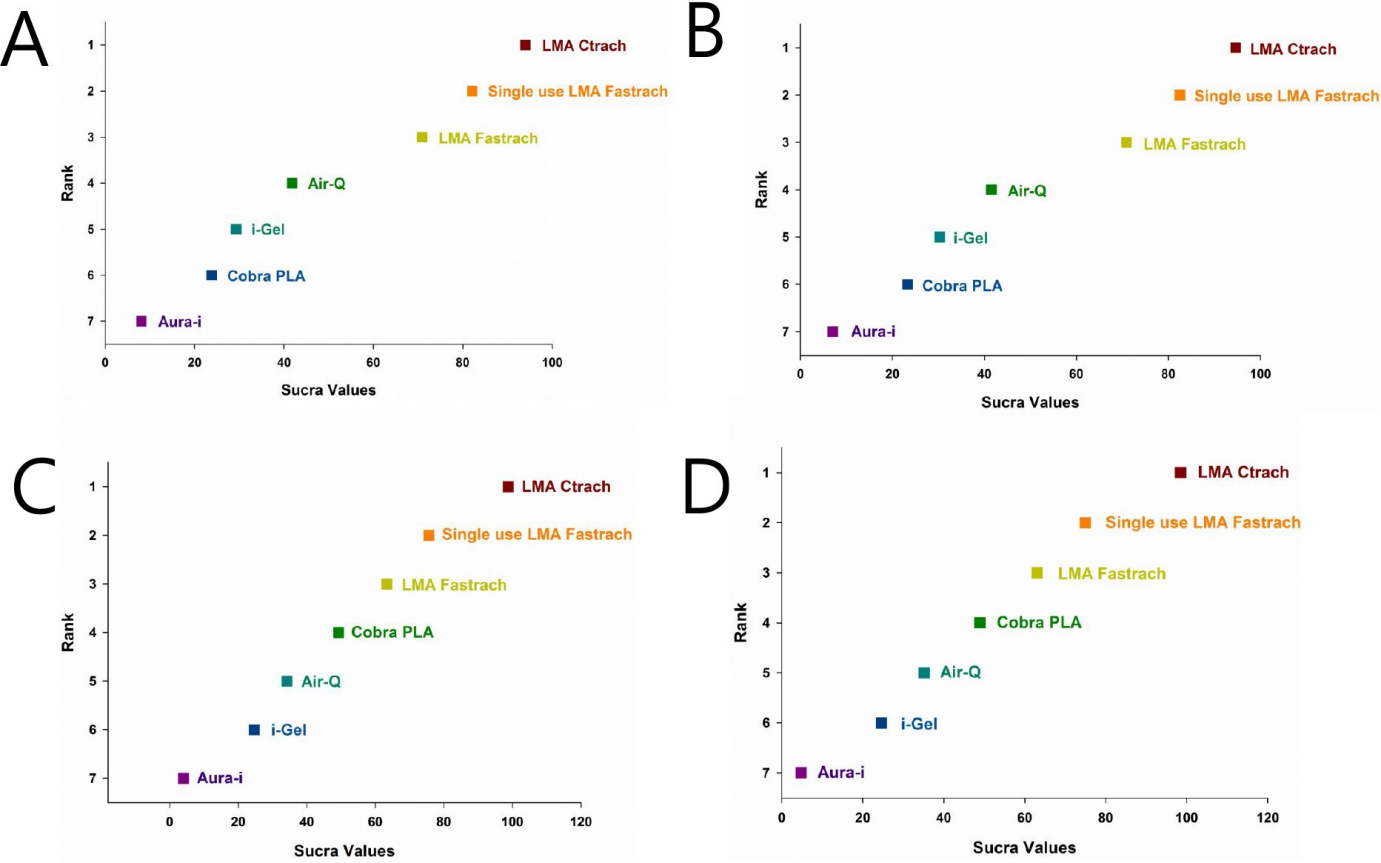
The expected ranking and SUCRA values of each airway device as a strategy for unassisted tracheal intubation. A. The overall success rate of unassisted tracheal intubation by ITT; B. The overall success rate of unassisted tracheal intubation by PP; C. The success rate at first attempt by ITT; D. The success rate at first attempt by PP. SUCRA, surface under the cumulative ranking curve; ITT, intention to treat; PP, per protocol.

For all outcomes of each specific datum, we presented the network plot, funnel plot, inconsistency plot, contribution plot, predictive interval plot, and SUCRA ranking. We also presented a summary of the results in a supplementary file. Fig 2 is a network plot showing the comparison of the overall success rate and success rate at first attempt among seven types of SGA devices as a strategy for tracheal intubation. Seven SGA devices were compared in the 16 RCTs (1 = single-use LMA-Fastrach, 2 = CobraPLA, 3 = Air-Q, 4 = LMA-Fastrach, 5 = i-gel, 6 = Ambu-Aura, and 7 = LMA-CTrach). The SUCRA probabilities of different SGA devices as strategies for unassisted tracheal intubation were calculated. The ranking of the cumulative probabilities for the SGA devices is shown in Fig 3. The expected ranking and SUCRA values of each airway device as a strategy for unassisted tracheal intubation are presented in Fig 4. The evaluation of network inconsistency by using the design-by-treatment interaction model is presented in “S1 Fig.” The contribution of each direct comparison of the estimation of the network summary effects is shown in “S2 Fig.” The estimated pair-wise summary effects are presented in “S3 Fig,” which shows the CI and PrI of the estimates. The comparison-adjusted funnel plots (“S4 Fig”) for all study outcomes were symmetrical around the zero line, which suggested a publication bias was less likely.

### A. Overall success rate of unassisted tracheal intubation

LMA-Fastrach and Air-Q were more frequently compared directly in both ITT and PP. LMA-Fastrach was the most frequent comparator across the RCTs (Fig 2A and 2B). Among the seven SGAs, LMA-CTrach was likely to be the most effective airway device as a strategy for unassisted tracheal intubation (Fig 3A and 3B). The three best SGA devices as a strategy for tracheal intubation were LMA-CTrach, single-use LMA-Fastrach, and LMA-Fastrach (Fig 4A and 4B). Air-Q ranked fourth (Fig 4A and 4B). Three closed loops were included in the network when comparing the overall success rate. All CIs for RoRs were compatible with zero inconsistency (RoR = 1). The evaluation of network inconsistency by using the design-by-treatment interaction model suggested no evidence of statistically significant inconsistency (*χ*^*2*^ (3) = 1.39, *P* = 0.707 for the overall success rate of unassisted tracheal intubation by ITT; *χ*^*2*^ (3) = 1.37, *P* = 0.712 for the overall success rate of unassisted tracheal intubation by PP) (“S1A and S1B Fig”). As shown in “S2A and S2B Fig,” two comparisons (single-use LMA vs. LMA-CTrach and LMA-Fastrach vs. Ambu-Aura) were presented using direct evidence alone. Seven comparisons (single-use LMA vs. CobraPLA, single-use LMA vs. air-Q, single-use LMA vs. LMA-Fastrach, single-use LMA vs. i-gel, CobraPLA vs. i-gel, air-Q vs. LMA-Fastrach, and LMA-Fastrach vs. i-gel) were presented using mixed evidence (both direct and indirect evidences). Finally, 18 comparisons of the overall success rate were presented using indirect evidence alone. The overall success rates of unassisted intubation by ITT and PP are shown in “S3A Fig” and “S3B Fig,” respectively. The comparison-adjusted funnel plots were symmetrical around the zero line, which suggested a publication bias was less likely (S4A and S4B Fig).

### B. Success rate at first attempt

LMA-Fastrach, Air-Q, i-gel, and single-use LMA-Fastrach were more frequently compared directly in both ITT and PP. LMA-Fastrach was the most frequent comparator across the RCTs (Fig 2C and 2D). Among the seven SGAs, LMA-CTrach was likely to be the most effective airway device as a strategy for unassisted tracheal intubation (Fig 3C and 3D). The three best SGA devices as a strategy for tracheal intubation were LMA-CTrach, single-use LMA-Fastrach, and LMA-Fastrach (Fig 4A and 4B). Cobra PLA ranked fourth (Fig 4C and 4D). Two closed loops were included in the network when comparing the success rate at first attempt by ITT. All CIs for RoRs were compatible with zero inconsistency (RoR = 1). The evaluation of network inconsistency by using the design-by-treatment interaction model suggested no evidence of statistically significant inconsistency (*χ*^*2*^ (2) = 1.48, *P* = 0.476 for the success rate at first attempt by ITT; *χ*^*2*^ (2) = 1.49, *P* = 0.474 for the success rate at first attempt by PP) (“S1C and S1D Fig”). As shown in “S2C and S2D Fig,” three comparisons (single-use LMA vs. air-Q, single-use LMA vs. Ambu-Aura, and LMA-Fastrach vs. LMA-CTrach) were presented using direct evidence alone. Five comparisons (single-use LMA vs. CobraPLA, single-use LMA vs. LMA-Fastrach, single-use LMA vs. i-gel, CobraPLA vs. i-gel, and LMA-Fastrach vs. i-gel) were presented using mixed evidence (both direct and indirect evidences). Finally, 14 comparisons of the success rate at first attempt were presented using indirect evidence alone. The overall success rates of unassisted intubation by ITT and PP are shown in “S3C Fig” and “S3D Fig,” respectively. The comparison-adjusted funnel plots were symmetrical around the zero line, which suggested a publication bias was less likely (S4C and S4D Fig).

### Sensitivity analysis

Among the 16 RCTs, only one study included pediatric patients[17]. The sensitivity analysis excluding this study showed no significant difference in the reported results.

## Discussion

In our NMA, LMA-CTrach was the best SGA device as a strategy for unassisted intubation. The fiberoptic system could be a major reason for this device being the best. LMA-CTrach has a functionally similar structure to that of LMA-Fastrach. Epiglottic down-folding is a main cause of failure of endotracheal tube advancement, which is as high as 80% during LMA-Fastrach insertion[40]. However, the fiberoptic system of LMA-CTrach is helpful in successful tracheal intubation as it enables visual confirmation via up-down maneuvers while maintaining ventilation to correct the positioning before tracheal intubation[33]. This system could prevent epiglottic down-folding by optimizing tube placement. However, even a small amount of secretion could impede the visual field, and repeated sterilization deteriorates the quality of the LMA-CTrach fiberoptics. The increased expense of LMA-CTrach relative to that of other SGA devices may also be a disadvantage.

Although LMA-CTrach and LMA-Fastrach are designed to be effective ventilatory devices and unassisted intubation guides in patients with normal and abnormal airways, these reusable silicone LMA devices have the potential for disease transmission through residual biological debris despite sterilization in the autoclave. Thus, polyvinyl chloride (PVC) single-use LMA devices have been introduced. In the study by Teoh and Lim, which compared PVC single-use LMA-Fastrach and conventional LMA-Fastrach, no significant differences were observed in the speed or success of unassisted intubation despite the difference in materials used[37].

Among the 16 RCTs evaluated in this review, only the study by Kleine-Brueggeney et al. included pediatric patients[17]. This study showed very low success rates with both Air-Q and Ambu-Aura (15% and 3%, respectively). Furthermore, Theiler et al. have suggested that SGA devices tend to perform worse in smaller children[41]. Because of epiglottic down-folding, unassisted intubation through an SGA device has the potential to cause injury to the epiglottis or glottis. Therefore, in pediatric patients, fiberoptic scope-guided intubation through an SGA device is recommended[17, 42, 43]. To rule out this influence, we performed a sensitivity analysis after excluding the Kleine-Brueggeney et al. study. However, no significant change was observed in the result, and Air-Q and Ambu-Aura were still ranked low.

Unassisted intubation is a common and helpful approach; however, it has been associated with serious complications and higher failure rates without the assistance of other devices within the larynx or pharynx to aid the visualization or direction of the endotracheal tube through the vocal cords[2, 3, 17, 44]. I-gel and Ambu-Aura ranked lowest in the success rate of unassisted intubation. The low success rate of i-gel seems to be closely related to the angle of the airway outlet. Although i-gel has a large airway outlet, the endotracheal tube gets easily caught in the arytenoid cartilage or other posterior structures of the larynx. Reshaping the outlet might be helpful for improving the success rate of tracheal intubation[8].

In our study, Ambu-Aura was a combination of Aura-I in the study by Kleine-Brueggeney et al[17]. and AuraGain in the study by Sethi et al[37]. Unlike Aura-I, AuraGain is a recently developed, third-generation SGA device that provides gastric control with intubating capability by using standard endotracheal tubes. Unfortunately, the study by Sethi et al[37]. is the first and only study using AuraGain. The lack of experience with AuraGain may have affected the results of that study. As we performed a sensitivity analysis after excluding the Kleine-Brueggeney et al. study, we could rule out the influence of combining two types of Ambu-Aura (Aura-I in Kleine-Brueggeney et al. and AuraGain in Sethi et al.). The result did not change significantly, with Ambu-Aura still being ranked low. Further RCTs are needed to evaluate newly developed SGA devices.

The insertion technique of endotracheal tubes may affect the success rate of unassisted intubation. In the Anuradha et al[28]. study, reverse orientation of the prewarmed conventional PVC endotracheal tube was used as the basis of the Kundra et al. study[9]. The prewarmed PVC endotracheal tube reduced the angle of emergence of the tube from the device and improved the success rate of intubation compared with that of silicone-reinforced tubes[9]. In the Darlong et al. study[29], lubrication of the PVC cuffed endotracheal tube before insertion through the CobraPLA decreased the resistance of dilating the space between the two medial-most bars of the CobraPLA by the endotracheal tube, which increased the success rate. Moreover, just before inserting the endotracheal tube through the CobraPLA, the removal of the 15-mm male connector made the effective length of the breathing tube shorter (by approximately 2 cm)[29]. However, the CobraPLA caused a higher incidence of trauma than did the LMA-Fastrach because of its stiffer head. In addition, while maneuvering the PVC endotracheal tube through the CobraPLA, it could be deflected from the grill and could strike against the pyriform fossa. However, our NMA did not consider these differences in endotracheal tube insertion technique, which may be a limitation of our research.

This study still has several limitations. First, the number of eligible RCTs was small. All results were significant only at the 95% CIs, not at the 95% PrIs. The lack of protocolled information size estimation before data analysis might affect the insufficient conclusion. Well-designed RCTs performed on large patient populations are required to increase the confidence of the results. Second, the estimates obtained from the NMA might be influenced by inconsistency in the NMA comparing more than two arms[45, 46]. A NMA compares multiple treatments by integrating both direct and indirect evidences into a general statistical frame work. Potentials that are estimated from indirect comparisons are less reliable than are those from direct comparisons, and inconsistency between direct and indirect evidences is one of the main issues[46]. Therefore, to improve the reliability of our results, we carefully reviewed the inconsistency of the entire network and loop, and the evaluation suggested no evidence of inconsistency. Third, reporting bias is a potential threat to the validity of the outcome of a NMA[47]. In our study, we ruled out the likelihood of publication bias by using comparison-adjusted funnel plots. Fourth, a dedicated wire-reinforced silicone endotracheal tube was advocated for intubation through LMA-Fastrach. The strategy of combining both LMA-Fastrach and this tube might affect the success rate of intubation. Therefore, the superiority of LMA-Fastrach could be the result of the concurrent use of LMA-Fastrach and the tube rather than LMA-Fastrach per se. Last, as shown in “S1 Table,” blinding of the investigators inserting the tracheal tube through the SGA devices was difficult or impossible to achieve. Although we exempted this parameter when scoring the risk of bias, the included RCTs had a high or uncertain risk of bias. Despite these limitations, the present NMA is the first to evaluate SGA devices. Moreover, in contrast to traditional meta-analysis (reporting pair-wise comparisons as the main results), the current NMA presented results by simultaneous clustered ranking for the success rate of unassisted intubation and demonstrated strength via a thorough review of consistency and sensitivity analysis.

## Conclusion

Sixteen RCTs comprising 2014 patients were included in this NMA to compare the effectiveness of SGA devices as a strategy for unassisted tracheal intubation. The types of SGA devices included were LMA-CTrach, LMA-Fastrach, Air-Q, i-gel, CobraPLA, Ambu-Aura, and single-use LMA. Based on the SUCRA, the three best SGA devices as a strategy for unassisted tracheal intubation are LMA-CTrach (which includes video-assisted tracheal tube guidance), single-use LMA-Fastrach, and LMA-Fastrach.

## Supporting information

S1 File Study protocol. (DOCX)

S2 File PRISMA NMA checklist of Items to include when reporting a systematic review involving a network meta-analysis. (DOCX)

S1 Table Risk of bias in the included studies. (DOCX)

S1 Fig Inconsistency plots of direct and indirect comparisons in this network meta-analysis. (DOCX)

S2 Fig Contribution plot for each direct comparison in the network. (DOCX)

S3 Fig The confidence intervals and predictive intervals of log estimates of the success rate of unassisted intubation. (DOCX)

S4 Fig Comparison-adjusted funnel plots. (DOCX)
