# Supplementary material for: Supraglottic airway devices as a strategy for unassisted tracheal intubation: A network meta-analysis
Source: PLoS One. 2018 Nov 5;13(11):e0206804. doi: 10.1371/journal.pone.0206804 (PMC6218066; doi:10.1371/journal.pone.0206804)
Supplement: S1 File — (DOCX) [file pone.0206804.s001.docx]

**S1 File. Study Protocol**

**Inclusion and Exclusion Criteria**

We included only randomised clinical trials that compared SGA devices as a strategy of unassisted tracheal intubation. Inclusion criteria were as follows: (1) randomised controlled trial (RCT); (2) patients who had surgery under general anesthesia using supraglottic airway devices as a strategy of tracheal intubation without use of fiberoptic bronchoscope as a guide; and (3) comparison between two different types of SGA devices group or more groups. Study outcomes including the overall success rate of tracheal intubation by the intention to treat (ITT) strategy as the primary outcome, and the overall success rate of tracheal intubation by the per protocol (PP) strategy and the success rate of tracheal intubation at first attempt by ITT and PP as secondary outcomes. The overall success rate of ITT was calculated by dividing the number of successful intubations (numerator) by the total number of patients (denominator). The overall success rate of PP was calculated by dividing the number of successful intubations (numerator) with the number of successful SGA device placements (denominator).

Exclusion criteria were as follows: 1) studies that only investigated SGA devices as a conduit of fibre optic-guided tracheal intubation; and 2) studies did not report outcome of interest.

There were neither language limitations nor date restrictions in our study.

**Information Sources and Search Strategy**

Two authors (HK and EJA) independently carried out database searches using MEDLINE, EMBASE, the Cochrane Central Register of Controlled Trials (CENTRAL), KoreaMed (<http://www.koreamed.org>) and Google Scholar databases to search for all relevant articles up to May 2017. The search strategy, which included a combination of free text, Medical Subject Headings (MeSH) and EMTREE terms, is described below.

**Medline**

1. randomized controlled trial.pt
2. randomized controlled trial$.mp
3. controlled clinical trial.pt
4. controlled clinical trial$.mp
5. random allocation.mp
6. exp double-blind method/
7. double-blind.mp
8. exp single-blind method/
9. single-blind.mp
10. or/1-9
11. clinical trial.pt
12. clinical trial$.mp
13. exp clinical trial/
14. (clin$ adj25 trial$).mp
15. ((singl$ or doubl$ or tripl$ or trebl$) adj25 (blind$ or mask$)).mp
16. random$.mp
17. exp research design/
18. research design.mp
19. or/11-18
20. 10 or 19
21. Case report.tw.
22. Letter.pt.
23. Historical article.pt.
24. Review.pt.
25. or/21-24
26. 20 not 25
27. exp intubation, intratracheal/
28. (intub$ or ((airway or respiratory tract) adj3 manage$)).mp.
29. or/27-28
30. 26 and 29
31. exp laryngeal masks/
32. laryngeal mask airway.mp.
33. LMA.mp.
34. (mask adj6 airway).mp.
35. (i-gel or i gel or igel).mp.
36. (air-Q or air q or airq).mp.
37. Aura.mp.
38. (cobra PLA or cobra perilaryngeal airway).mp.
39. Laryngeal tube.mp.
40. Combitube
41. or/31-40
42. 30 and 41

**EMBASE**

1. randomi?ed controlled trial$.mp.
2. 'controlled clinical trial (topic)'/exp
3. controlled AND clinical AND trials
4. controlled clinical trial$.mp.
5. 'randomization'/exp
6. 'random allocation'/exp
7. random allocation.mp.
8. double-blind.mp.
9. single-blind.mp.
10. #1 OR #2 OR #3 OR #4 OR #5 OR #6 OR #7 OR #8 OR #9
11. 'clinical trial (topic)'/exp
12. clinical AND trial$.mp.
13. random$.mp.
14. rct
15. #11 OR #12 OR #13 OR #14
16. #10 OR #15
17. 'case study'/exp
18. 'case report'/exp
19. 'abstract report'/exp
20. 'letter'/exp
21. #17 OR #18 OR #19 OR #20
22. #16 NOT #21
23. ‘respiratory tract intubation’/exp
24. Intubation.mp.
25. #23 OR #24
26. #22 AND #25
27. 'laryngeal mask'/exp
28. laryngeal mask airway.mp.
29. 'lma'/exp
30. i-gel or i gel or igel.mp.
31. air-Q or air q or airq.mp.
32. Aura.mp.
33. Cobra PLA
34. cobra perilaryngeal airway
35. Laryngeal tube
36. Combitube
37. #27 OR #28 OR #29 OR #30 OR #31 OR #32 OR #33 OR #34 OR #35 OR #36
38. #26 AND #37
