# Supplementary material for: Supraglottic airway devices as a strategy for unassisted tracheal intubation: A network meta-analysis
Source: PLoS One. 2018 Nov 5;13(11):e0206804. doi: 10.1371/journal.pone.0206804 (PMC6218066; doi:10.1371/journal.pone.0206804)
Supplement: S1 Table — (DOCX) [file pone.0206804.s003.docx]

S1 Table. Risk of bias in the included studies

| **Biases/ References** | **Random sequence generation** | **Allocation concealment** | **Blinding of outcome assessment** | **Incomplete outcome data** | **Selective reporting** | **Other bias** | **Overall risk of bias** |
| --- | --- | --- | --- | --- | --- | --- | --- |
| **Anuradha 2017** | Low risk | Unclear | Unclear | Low risk | Low risk | Low risk | Unclear |
| **Darlong 2011** | Low risk | Unclear | Unclear | Low risk | low risk | Low risk | Unclear |
| **Erlacher 2011** | Low risk | Unclear | Unclear | Low risk | Low risk | Low risk | Unclear |
| **Garzon 2014** | Low risk | Unclear | Unclear | Low risk | Low risk | Low risk | Unclear |
| **Halwagi 2012** | Low risk | Unclear | Unclear | Low risk | Low risk | Low risk | Unclear |
| **Kapoor 2014** | Low risk | Unclear | Unclear | Low risk | Low risk | Low risk | Unclear |
| **Karim 2011** | Unclear | Low risk | Unclear | Low risk | Low risk | Low risk | High risk |
| **Kleine 2015** | Low risk | Low risk | Unclear | Low risk | Low risk | Low risk | Low risk |
| **Liu 2008** | Low risk | Unclear | Unclear | Low risk | Low risk | Low risk | Unclear |
| **Malhotra 2016** | Low risk | Unclear | Unclear | Low risk | Low risk | Low risk | Unclear |
| **Neoh 2012** | Low risk | Low risk | Unclear | Low risk | Low risk | Low risk | Low risk |
| **Sastre 2012** | Low risk | Low risk | Unclear | Low risk | Low risk | Low risk | Low risk |
| **Sethi 2017** | Low risk | Low risk | Unclear | Low risk | Low risk | Low risk | Low risk |
| **Teoh 2007** | Low risk | Low risk | Unclear | Low risk | Low risk | Low risk | Low risk |
| **Theiler 2011** | Low risk | Low risk | Unclear | Low risk | Low risk | Low risk | Low risk |
| **Yang 2013** | Low risk | Unclear | Unclear | Low risk | Low risk | Low risk | Unclear |
| Method of estimating overall risk of bias: If all results or the above items were “low risk”, the overall risk of bias of the trial was deemed to be low risk of bias. If more than one of the above items were “unclear” or “high risk”, the overall risk of bias of the trial was deemed to be unclear risk of bias or high risk of bias, respectively. High risk indicates high risk of bias; low risk, low risk of bias; unclear risk, unclear risk of bias because of lack of detailed reports. | | | | | | | |
