# Supplementary material for: Supraglottic airway devices as a strategy for unassisted tracheal intubation: A network meta-analysis
Source: PLoS One. 2018 Nov 5;13(11):e0206804. doi: 10.1371/journal.pone.0206804 (PMC6218066; doi:10.1371/journal.pone.0206804)
Supplement: S1 Fig — (DOCX) [file pone.0206804.s004.docx]

**S1 Fig. consistency plots**


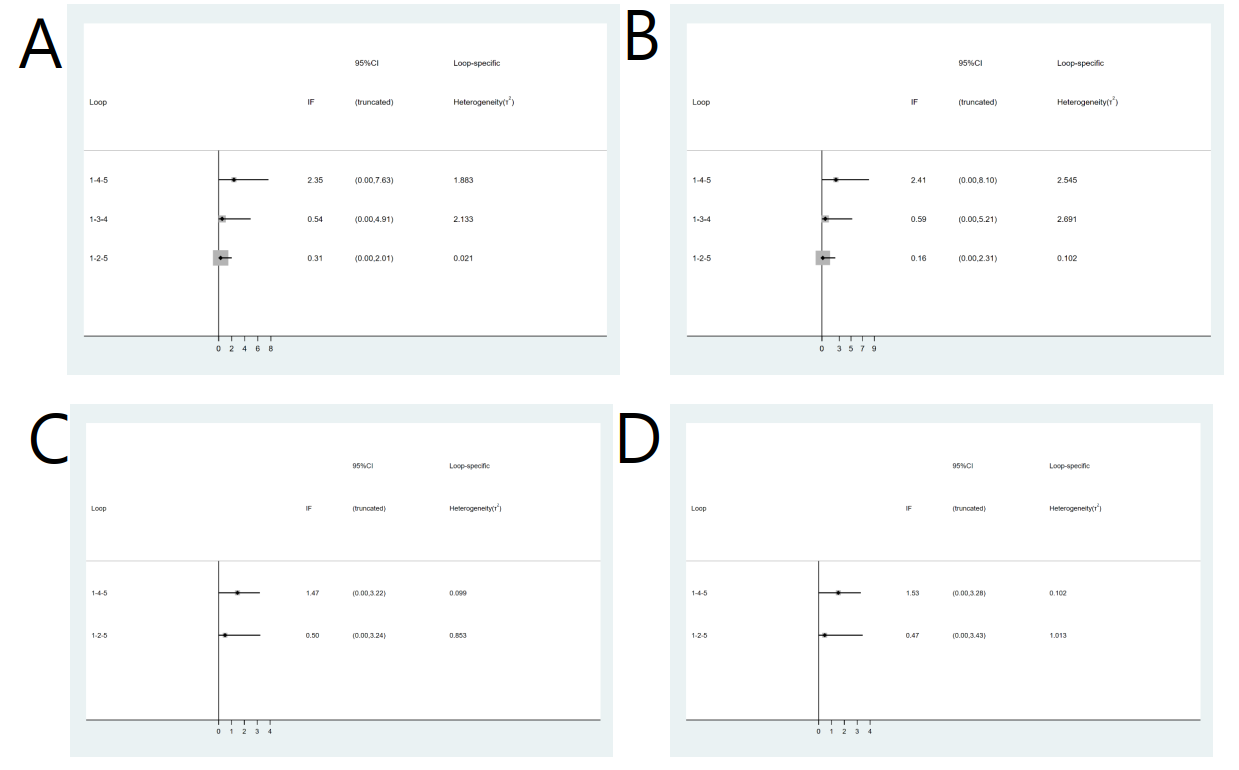


S1 Fig. Inconsistency plots of direct and indirect comparisons in this network meta-analysis. A. Overall success rate of unassisted intubation by ITT; B. Overall success rate of unassisted intubation by PP; C. Success rate of first attempt by ITT; D. Success rate of first attempt by PP.

ITT, intention to treat; PP, per protocol.
