# Supplementary material for: Supraglottic airway devices as a strategy for unassisted tracheal intubation: A network meta-analysis
Source: PLoS One. 2018 Nov 5;13(11):e0206804. doi: 10.1371/journal.pone.0206804 (PMC6218066; doi:10.1371/journal.pone.0206804)
Supplement: S2 Fig — (DOCX) [file pone.0206804.s005.docx]

**S2 Fig. Contribution plots**


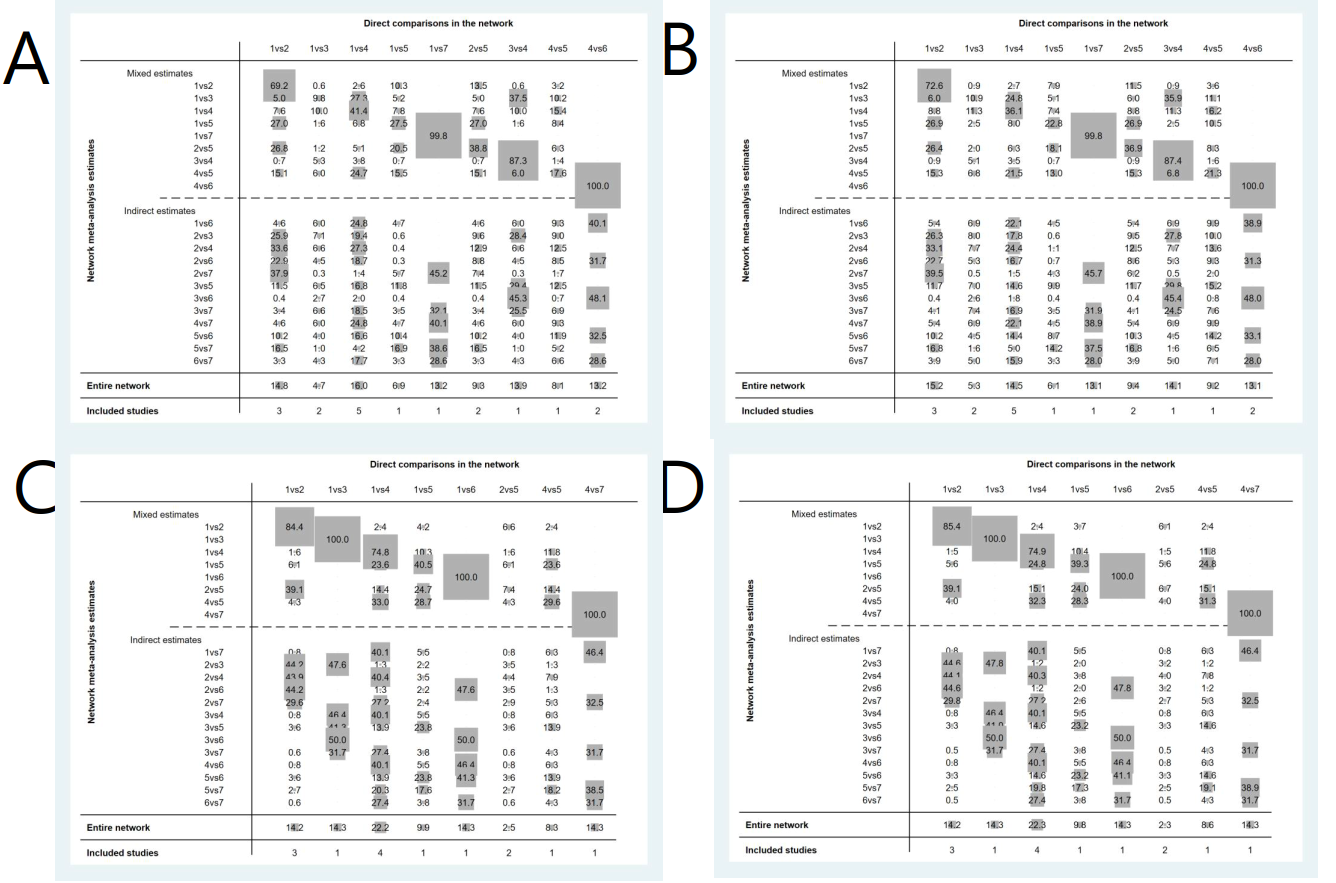


S2 Fig. Contribution plot for each direct comparison in the network. Rows correspond to mixed and indirect evidence and columns correspond to direct evidence. Percentage contribution of each direct comparison to the network summary estimates and in the entire network. The sizes of the boxes are proportional to the percentage contribution of each direct estimate to the network meta-analysis estimates (rows 1–8) and to the entire network. The last row shows the number of included direct comparisons. A. Overall success rate of unassisted intubation by ITT; B. Overall success rate of unassisted intubation by PP; C. Success rate of first attempt by ITT; D. Success rate of first attempt by PP. ITT, intention to treat; PP, per protocol
