# Supplementary material for: Supraglottic airway devices as a strategy for unassisted tracheal intubation: A network meta-analysis
Source: PLoS One. 2018 Nov 5;13(11):e0206804. doi: 10.1371/journal.pone.0206804 (PMC6218066; doi:10.1371/journal.pone.0206804)
Supplement: S3 Fig — (DOCX) [file pone.0206804.s006.docx]

**S3 Fig. Predictive interval plots**


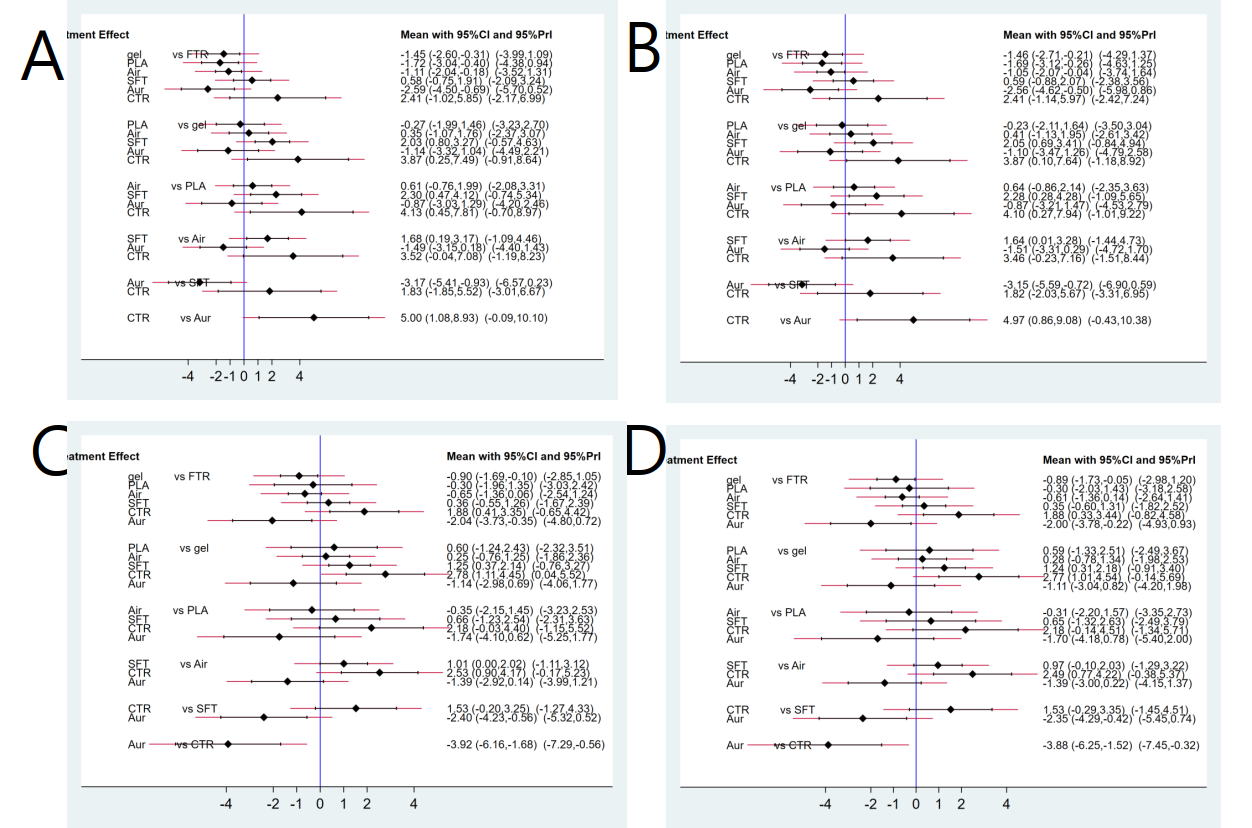


S3 Fig. The confidence intervals and predictive intervals of log estimates of the success rate of unassisted intubation. Black corresponds to 95% confidence interval and red corresponds to 95% predictive interval. A. Overall success rate of unassisted intubation by ITT; B. Overall success rate of unassisted intubation by PP; C. Success rate of first attempt by ITT; D. Success rate of first attempt by PP. ITT, intention to treat; PP, per protocol
