# Supplementary material for: Supraglottic airway devices as a strategy for unassisted tracheal intubation: A network meta-analysis
Source: PLoS One. 2018 Nov 5;13(11):e0206804. doi: 10.1371/journal.pone.0206804 (PMC6218066; doi:10.1371/journal.pone.0206804)
Supplement: S4 Fig — (DOCX) [file pone.0206804.s007.docx]

**S4 Fig. Comparison-adjusted funnel plots**


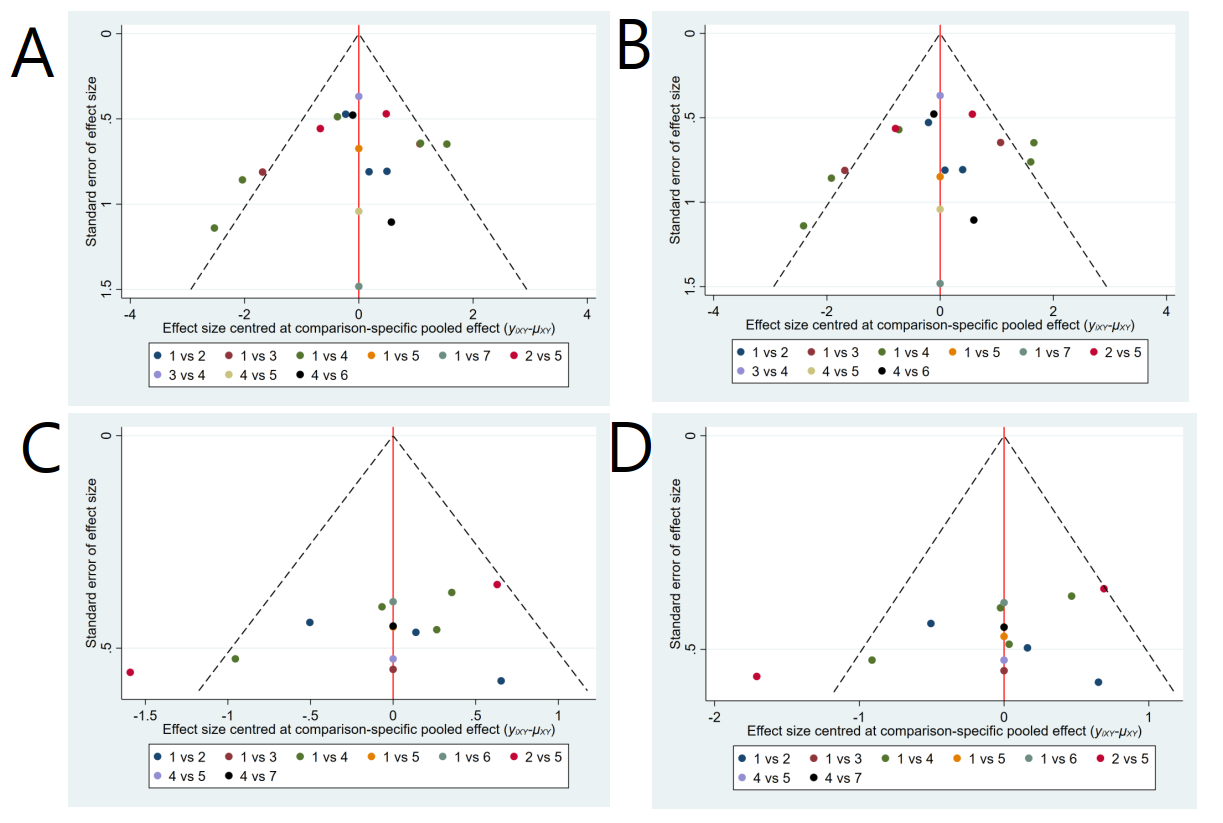


S4 Fig. Comparison-adjusted funnel plots. A. Overall success rate of unassisted intubation by ITT; B. Overall success rate of unassisted intubation by PP; C. Success rate of first attempt by ITT; D. Success rate of first attempt by PP. ITT, intention to treat; PP, per protocol. 1=single-use LMA-Fastrach; 2=CobraPLA; 3=Air-Q; 4=LMA-Fastrach; 5=i-gel; 6=Ambu-Aura; 7=LMA-CTrach
